# Chemistry, manufacturing, and controls for live microbial products: addressing classification challenges and enhancing process validation

**DOI:** 10.3389/frmbi.2025.1569348

**Published:** 2025-06-27

**Authors:** Ralph Slijkerman, Rob van Dijk, Nasser Mohieddin, Ivana Ciarlante, Antal de Jong, Moira Monika Schuler

**Affiliations:** ^1^ Department of Manufacturing Science and Technology (Production), Wacker Biotech B.V., Amsterdam, Netherlands; ^2^ Client and Contract Management Department, Wacker Biotech B.V., Amsterdam, Netherlands; ^3^ Client and Contract Management Department, Wacker Biotech US, San Diego, CA, United States; ^4^ BioProcess Development Department, Wacker Biotech B.V., Amsterdam, Netherlands

**Keywords:** live microbial product (LMP), injection, regulatory, classification, live biotherapeutic product (LBP), live bacterial therapeutic (LBT), monoseptic, commercialization

## Abstract

Traditional process validation life cycles need to be tailored to the specific needs of live microbial products (LMPs). LMPs can be divided into subcategories, and the product characteristics are the basis for the regulatory category and thereby the applicable guidelines. All LMPs fall under regulations related to GMP-compliant manufacturing; however, there are live microbial specific challenges. Both the FDA and the EMA do not have a regulatory framework for LMPs administered by injection. Full adherence to general guidelines for injectables is technically not feasible for LMPs, as sterility is required, which stands in conflict with living organisms as a product. Safety-related critical quality attributes (CQAs) of such LMPs typically include the absence of contaminants and proof of monoseptic condition of the product. This paper aims to holistically outline and compare LMP-relevant guidelines while highlighting different subcategories. Additionally, the status of the field is captured by collecting all LMP-related clinical trials to resolve specific challenges in LMP development. Taken together, this overview will aid in bringing future LMPs from development to commercialization.

## 1 Introduction

To quote Cordaillat-Simmons and colleagues, "Regulatory and market success for a live biotherapeutic product (LBP) will depend on the quality of the development involving the credible demonstration of safety and efficacy in the intended population" (Cordaillat-Simmons et al., 2020). Beyond the aspect of clinical trials aimed at demonstrating safety and efficacy, it is crucial, as with any other finished product, to demonstrate quality—in terms of "ensuring and providing documentary evidence that processes (within their specified design parameters) are capable of consistently producing a finished product of the required quality" (European Medicines Agency, 2016). To demonstrate that processes can consistently produce products of the required quality, it is essential to pair the appropriate analytical panel with effective process development and characterization for live microbial products (LMPs). This approach ensures a robust and consistent manufacturing process, which is ultimately validated through a successful process performance qualification, as is expected for any biological medicinal product.

**Figure 1:**
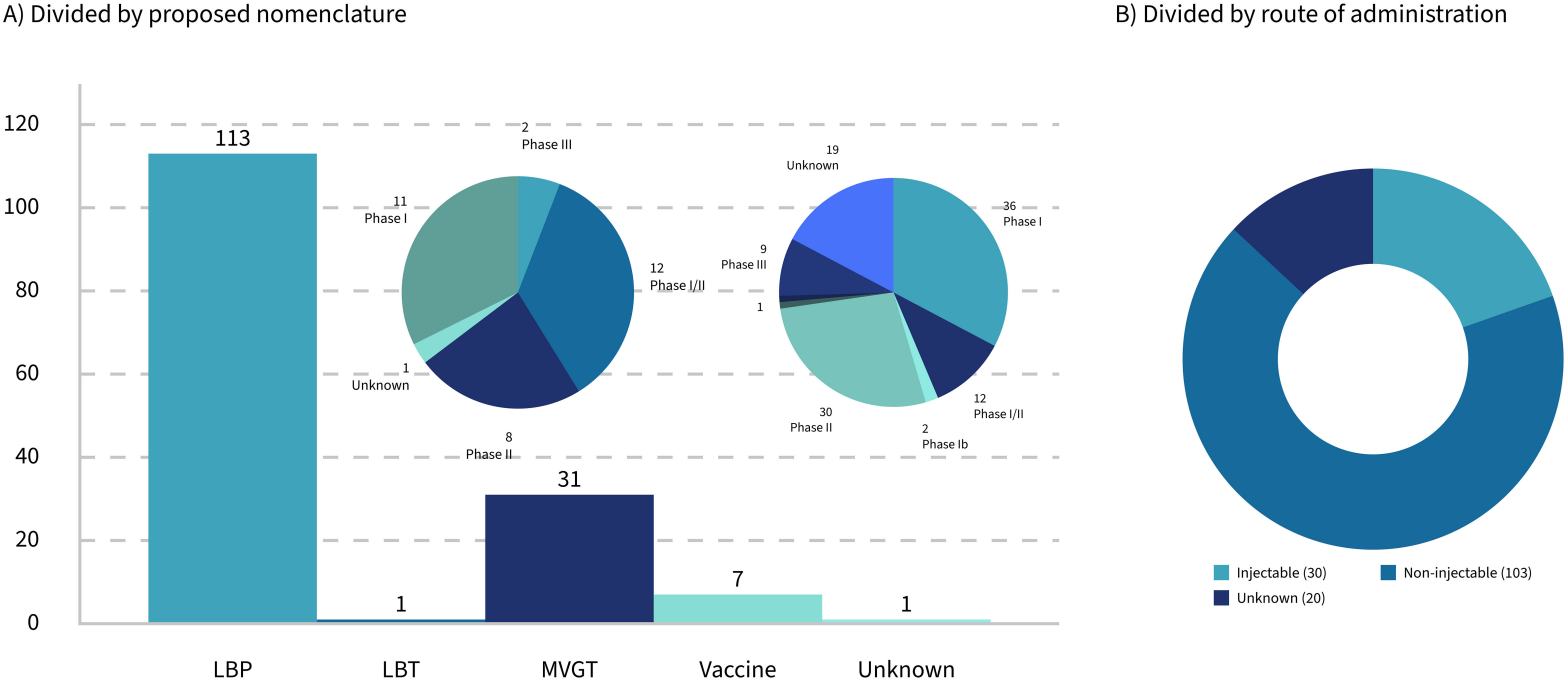
Breakdown of 153 LMP clinical trials by nomenclature **(A)** and delivery methods **(B)**.

This paper will focus on how the traditional process validation life cycle for biological products needs to be tailored to the specific needs of LMPs used as drugs, including, but not limited to, LBPs, for successful market access in Europe and the United States. First, stakes will be taken regarding the currently approved live microbial products, and the ambiguities in the regulatory landscape surrounding drugs consisting of living microbials (Microbiome Therapeutics Innovation Group and Barberio, 2024) will be highlighted. Special emphasis will be given to LMPs administered by injection, which do not fall under the umbrella of the LBP framework but seem to get traction when looking at the clinical trials that are currently ongoing. Of all the trials with a known route of administration, 22.5% are identified as injectables (**Figure 1B**). Then, the basics of process validation (for biologics) will be summarized, before focusing on the specific needs for those LMPs administered by injection. The paper will address the question of whether the regulatory category of a specific LMP has an impact on the development and validation of the associated manufacturing processes of those life-saving "bugs as drugs" that are currently in the clinical pipeline.

### 1.1 Important milestones in terms of market access for live microbial products

An important year for the live microbial world was 2022, as Rebyota and Vowst (formerly SER-109) were approved by the U.S. Food and Drug Administration (FDA) as the first two LBPs for commercial use. Rebyota is a live, fecal microbiota administered via rectal enema, and Vowst is an orally taken capsule of live microbiota spores. These first two products reached the market approximately 10 years after the FDA provided the first guideline for LBPs.

While classified as LBPs in several publications (Monday et al., 2024), both Rebyota and Vowst are often also called fecal microbial transplants (FMTs). There is an expectation that more such LBPs or FMTs will receive market authorization soon, given that in 2020, there were 134 active clinical trials involving such donor-derived LBPs or FMTs, of which 18 FMTs were in clinical phase III (Servetas et al., 2022).

Vowst differs from other FMTs because it is an oral, capsular formulation of approximately 50 species of Firmicutes spores (Jain et al., 2023) compared to the traditional naso-jejunal tube, colonoscopy, or retention enema approaches.

Compared to Vowst and Rebyota, which are both donor-derived products, the so-called "designed consortia" rely on a specific combination of (commensal) strains (McChalicher and Auniņš, 2022). A good example is Vedanta Biosciences' LBP product, consisting of eight strains of non-toxic, non-pathogenic *Clostridia* manufactured from clonal cell banks (Louie et al., 2023).

### 1.2 The origin of live microbial products

Before the FDA was even established, scientists Busch and Coley experimented with the use of *Serratia* and *Streptococcus* strains in the fight against terminal cancer in the late 1800s, sometimes with fatal outcomes (Brevi and Zarrinpar, 2023). Beyond this example, the use of FMTs dates back over 2,000 years according to certain sources (Monday et al., 2024).

Since 1989, 27 countries have been administering the FDA-approved Ty21a (typhoid vaccine live oral) Vivotif^®^ (Ty21a), and in June 2016, the FDA approved CVD 103-HgR (VAXCHORA™), a single-dose, oral cholera vaccine containing an attenuated form of *Vibrio cholerae* developed by SSVI Bern and eventually marketed by Pax-Vax (Levine et al., 2017). With this background, one can argue that neither Rebyota nor Vowst has been the first two LBPs to be approved by the FDA, and given the definition of an LBP by the FDA that specifically excludes vaccines, one can even argue that none of the abovementioned drug products qualify as LBPs.

### 1.3 Exploring the niches of bugs as drugs

Given the ambiguity around the terminology for LMPs, it is crucial to start with the definition of this niche category of medicinal products that is composed of a multitude of niche products. There are vaccines based on live organisms: traditional, orally administered LMPs; other engineered LMPs; microbiome- or microbiota-based products: delivery GMOs; and sometimes even pharmabiotics—basically a whole cohort of products that can be defined as "bugs as drugs" or LMPs as they will be named throughout this article. It seems that in this niche market of LMPs, every single entity has invented a new name for their type of product and maybe rightfully so, as every LMP seems to have a different approach, a different mode of action, a different approach with regard to CMC activities, and hence a distinct approach to regulatory approval and commercialization.

With the approval of the first "LBPs," the path to market access seems set, except that it is not. The approval of the first LBP highlights just more than ever that there is no clear regulatory framework for LMPs in general, just as there is no clear classification or commonly accepted nomenclature. There are a few guidelines that are specific to LMP categories, and there is a huge mesh of gray areas in between the different regulations, which might or might not apply to LMPs.

## 2 Live microbial products: regulatory landscape, clinical trials, and CMC-related aspects

### 2.1 Categorizing live microbial products

Any product that contains living microbials can be categorized by the broad term "live microbial product" (LMP—**Table 1**), which includes products intended as food, food supplement, or drug. Despite the regulatory guidelines related to LMPs, which focus on more specific product types, in this paper, we will focus on products used as a human drug. These are also sometimes referred to as pharmabiotics (Franciosa et al., 2023) or microbiome-based medicinal products (MMPs) (Rodriguez et al., 2025). MMPs or pharmabiotics differ from other live microorganism-based products, such as probiotics. The latter are used for several other purposes, such as (medical) food or derivatives (supplements) or cosmetics; therefore, topics related to probiotics are purposely excluded from the scope of this paper.

**Table 1:** Description of terms and abbreviations.

Term	Abbreviation	Description	Manufacturing under GMP required
Fecal microbial transplant	FMT	Group of products related to the screening, collection, storage, and administration of fecal material	No, but SoHO regulation applies^1^
Live bacterial therapeutic	LBT	Products consisting of living organisms, which are not orally administered (like LBPs) and are not engineered (like MVGTs)	Yes
Live biotherapeutic product	LBP	Product that contains live organisms and is applicable to the prevention, treatment, or cure of a disease or condition of human beings, but is not a vaccine. In addition, an LBP should not be administered by injection and is not an oncolytic bacteria	Yes
Live microbial product	LMP	Term overarching all prescriptive and non-prescriptive drugs consisting of living microorganisms	No
Microbial vector for gene therapy	MVGT	Products consisting of genetically modified microorganisms	Yes
Microbiome-based therapy	MBT	Therapy to reshape the composition of resident microbial communities and thereby restore health (https://www.frontiersin.org/journals/immunology/articles/10.3389/fimmu.2022.1046472/full) (Md Zahidul et al., 2023)	Yes^2^
Microbiome-based medicinal products	MMP	Medicinal products containing live microbial organisms (bacterial or yeasts) for human use (Rodriguez et al., 2025)	Yes^2^
Microbiota transplants	MT	See FMT	No
Next-generation probiotics	NGP	Products containing strains isolated from the human gut, showing potential health benefits based on comparative analyses (Martin and Langella, 2019)	No
Pharmabiotics	N/A	Products consisting of living microorganisms with a medical claim aiming to address or prevent specific medical conditions	No
Postbiotics	N/A	Probiotic-derived biologically active metabolites	No
Prebiotics	N/A	Foods that act to simulate the existing human microflora	No
Probiotics	N/A	General term for non-prescriptive products containing living microorganisms associated with a health claim and includes a.o., synbiotics, and postbiotics (1)	No
Synbiotics	N/A	Food ingredients and dietary supplements combining probiotics and prebiotics	No

^1^SoHO regulation: "Regulation on standards of quality and safety for substances of human origin intended for human application" (2024).

^2^Exception can apply. See section 2.1.

There is a key distinction between probiotics and pharmabiotics: probiotics are associated with a "health claim," while pharmabiotics are linked to a "medicinal claim" (Pot and Vandenplas, 2021; Grilc et al., 2023). In essence, probiotics focus on promoting general health benefits, whereas pharmabiotics aim to address or prevent specific medical conditions (Cordaillat-Simmons et al., 2020). In the United States, both types of microbial products—those with health claims and those with medical claim—are evaluated and regulated by the FDA, albeit under different guidelines. In Europe, however, the regulatory framework differs, as probiotics with health claims fall under the jurisdiction of the European Food Safety Authority (EFSA), while pharmabiotics with medicinal claims are overseen by the European Medicines Agency (EMA) (Pot and Vandenplas, 2021; Cordaillat-Simmons et al., 2020).

Another relevant category, which is on the edge of the definitions, is fecal microbiota transplants (FMTs). FMT is a group of products related to the screening, collection, storage, and administration of fecal material. This can be derived from both a direct donor and through centralized manufacturing from cell banks. FMTs are defined differently and follow different guidelines per country, which can make it confusing in some cases whether a product is qualified as FMT or LBP. One such example is the approved products from Ferring (Rebyota) and Seres (Vowst). These products are derived from qualified stool donors, but they are qualified as LBP. The reason is that they follow a standardized manufacturing process under good manufacturing practices (GMP) methods and are thoroughly screened for the absence of at least 29 pathogens (according to FDA requirements). Drugs derived from stool donors, which are not characterized, follow an unstandardized manufacturing process and/or study design and therefore are classified as FMT following guidance according to safety for organs and substances of human origin (SoHO) (Gonzales-Luna and Tillotson, 2023). Hence, the method of manufacturing and the associated level of control determine the classification of the final drug, rather than its application or characteristics. Due to different manufacturing processes and specificities of regulation around FMTs compared to other LMPs, FMTs that are not considered as LBPs will be out of scope for this paper. A detailed overview of the spectrum of microbiome-based therapies and MTs (microbiota transplants), as well as FMTs in particular, has recently been published by Rodriguez et al. (2025).

Conventional probiotics consist of lactic acid bacteria or bifidobacteria or a combination of those two, whereas next-generation probiotics (NGPs) consist of strains that are identified based on comparative microbiota analysis, showing health benefits. The following strains are commonly used as NGPs: *Akkermansia muciniphila*, *Faecalibacterium prausnitzii*, *Eubacterium hallii*, and *Roseburia* spp (Martin and Langella, 2019; Huanchang et al., 2022). With probiotics, NGPs, and FMTs, or MTs as more recently referred to (Rodriguez et al., 2025) set aside, LMPs can be separated into four main categories (**Table 2**): LBPs, MVGTs, vaccines, and LBTs.

**Table 2:** Overview of different live microbial product types and relevant guidelines in the EU and US applicable for these types of products (V = covered by the guideline, X = not covered by the guideline).

	LMP				
	LBP	MVGT	LBT	Vaccines	Others (e.g., probiotics)
Properties					
Live/inactivated microbials	Live	Both	Both	Both	Live
Engineered	Both	Yes	No	Both	Both
Route of administration	Non-injectable	Injectable + non-injectable	Injectable	Injectable + non-injectable	Non-injectable
Guidelines					
FDA LBP guideline^1^	V	V (if under LBP definition)	X	X	V (if falling under LBP definition)
FDA MVGT guideline^2^	V (if also falling under MVGT)	V	X	X	V (if falling under MVGT)
FDA guidance environmental assessment for microbial products^3^	V	V	V	V	V
EudraLex Volume 4, Annex I^4^	X	V (if injectable)	V	V (if injectable)	X
Directive 2001/83/EC in the EU^5^	V	V	V	V	X
EMA ATMP^6^	V (if genetically modified)	V	X	V (if genetically modified strain)	X
EMA Monograph on Live Biotherapeutic Products^7^	V	V	V	V	X
ICH Q11	V	V	V	V	V
FDA Guidance for Industry – Process Validation	V	V	V	V	V

1. FDA (CBER) Early Clinical Trials With Live Biotherapeutic Products: Chemistry, Manufacturing, and Control Information (2016) (https://www.fda.gov/files/vaccines,%20blood%20%26%20biologics/published/Early-Clinical-Trials-With-Live-Biotherapeutic-Products–Chemistry–Manufacturing–and-Control-Information–Guidance-for-Industry.pdf).

2. Recommendations for Microbial Vectors used for Gene Therapy (September 2016 (https://www.fda.gov/files/vaccines,%20blood%20&%20biologics/published/Recommendations-for-Microbial-Vectors-Used-for-Gene-Therapy–Guidance-for-Industry.pdf).

3. FDA's guidance (CBER) "Determining the Need for and Content of Environmental Assessments for Gene Therapies, Vectored Vaccines, and Related Recombinant Viral or Microbial Products.

4. EudraLex Volume 4, Annex I (https://health.ec.europa.eu/document/download/e05af55b-38e9-42bf-8495-194bbf0b9262_en?filename=20220825_gmp-an1_en_0.pdf).

5. https://eur-lex.europa.eu/legal-content/ENG/TXT/PDF/?uri=CELEX:32001L0083.

6. https://www.ema.europa.eu/en/human-regulatory-overview/advanced-therapy-medicinal-products-overview/guidelines-relevant-advanced-therapy-medicinal-products

7. European Pharmacopoeia 3053E General monograph on Live Biotherapeutic Products for human use (EDQM, 2019).

8. ICH Q11 Development and manufacture of drug substances (chemical entities and biotechnological/biological entities) - Scientific guideline, 2012.

9. FDA Guidance for Industry – Process Validation: General Principles and Practices, R1, published January 2011.

A live biotherapeutic product (LBP) is an official category which is widely used and defined in the FDA guideline: "*Early Clinical Trials with Live Biotherapeutic Products: Chemistry, Manufacturing, and Control Information*" (Food and Drug Administration, 2016), first published in 2012, as a product that contains live organisms and is applicable to the prevention, treatment, or cure of a disease or condition of human beings, but is not a vaccine. In addition, an LBP should, as a general matter, not be administered by injection and is not an oncolytic bacterium. Since 2019, the European Directorate for the Quality of Medicines & HealthCare (EDQM), cooperating with the EMA, has provided LBP-specific quality guidance in a dedicated monograph: "*3053E General Monograph on Live Biotherapeutic Products*" (European Pharmacopoaei, 2019). Ph. Eur. monograph 3053 specifically focuses on the production method including the removal of impurities or adventitious agents and on the used microorganism and its characterization (Franciosa et al., 2023).

Drugs that are used as a microbial vector for gene therapy (MVGT) are described as a separate category, with their own guideline for the US market (Food and Drug Administration, 2016). Whereas natural LMPs are ideal for general health applications and conditions where naturally occurring strains are sufficient, the engineered category differs from other categories, as the microbial product is genetically modified, typically to deliver some kind of payload for some form of cancer therapy. Additionally, other complex diseases, such as autoimmune disorders or metabolic diseases, can be targeted with genetically modified organisms (GMOs) to offer greater flexibility and precision. However, GMOs may raise safety concerns, such as the potential for horizontal gene transfer and unintended off-target effects or immune responses. Hence, a more extensive safety testing of the organism compared to natural strains is required, as well as additional CMC activities such as a strict containment and the need to confirm retention of the genetic modifications, ensuring stability. Thus, the development of genetically engineered LMPs is generally more complex and expensive than naturally occurring LMPs, and the general concerns about GMOs might shape public perception and foster greater resistance to adoption. Nevertheless, GMO LMPs offer unparalleled potential for treating complex diseases and conditions that require targeted or engineered solutions.

Following the LBP definition, vaccines consisting of live (attenuated) strains form a separate group entirely, where different guidelines apply for injectable or other administration forms of these vaccines.

There is a remaining group of drug products that are officially not categorized. This group consists of living organisms that are not orally administered (unlike LBPs) and are not engineered (otherwise they fall under the MVGT definition) and, in general, are not a vaccine. This group of products is here classified as live bacterial therapeutics (LBT).

According to the FDA definition, an LBP should contain living microbial organisms. For all other categories, the product can also contain inactivated microbials. In such cases, the word "live" does not apply. Manufacturing process steps are similar for products containing live or inactivated microbials, with an inactivation step as an additional activity for the latter product group. For the analytics, there are some minor differences between a product containing live or inactivated microbials, for instance, the need for an "inactivation assay" confirming the inactivation of the microbe prior to the final formulation.

Although single- and multistrain products differ from an analytical and manufacturing perspective, regulatory frameworks do not distinguish between them. As a result, this characteristic is not factored into the classification of microbial products.

To summarize the general approach to categorization of all different microbial products, distinctions are made based on five main criteria: 1) the level of control over the manufacturing process (distinguishing FMTs from other LMPs), 2) the physiological state of the microorganism (live or inactivated), 3) the genotype of the microorganism (engineered or wild-type strain), 4) the route of administration (oral, topical, or by injection), and 5) the overarching application (therapeutic or preventive). As for any other (investigational) medicinal product, the latter two categories will determine which regulatory guidelines apply for the development, manufacturing, and finally market authorization as we will see below.

### 2.2 Current regulatory landscape

Now that the properties of the different products as well as the nomenclature have been clarified (Section 2.1), it is important to look at the regulations and regulatory guidelines that are distinctive for each category (**Table 2**).

As illustrated in **Table 2**, there is no overarching guidance document encompassing all categories of LMPs, except for the general principles and practices that apply to the manufacturing of any medicinal product (Food and Drug Administration, 2016; European Medicines Agency, 2016). A genuine FMT product must adhere to the "Enforcement Policy Regarding Investigational New Drug Requirements for Use of Fecal Microbiota" for the US market and the so-called SoHO guideline (European Union, 2024) for the European market. In contrast, donor-derived LBPs (such as Rebyota and Vowst) are primarily governed by the "Early Clinical Trials With Live Biotherapeutic Products" guideline in the US and the "3053E General Monograph on Live Biotherapeutic Products" in the European Union.

Vaccines, whether they are orally or otherwise administered, are covered by the Code of Federal Regulation (Title 21) and approved by the FDA. In Europe, both are regulated via EudraLex Volume 4. In the first case, they will need to comply with Annex II and in the latter with Annex I (European Commission, 2022).

Engineered LMPs fall under the guidelines for MVGT in the United States. Furthermore, in specific cases, the LBP guideline might apply as well. In Europe, the situation is slightly different. Depending on the route of administration, the Directive 2001, EMA Monograph, and/or EudraLex Volume 4, Annex I might apply.

In any case, as stated above, for both vaccines and engineered LMPs, the Code of Federal Regulation is applicable for products for the US market. In Europe, EudraLex Volume 4, Annex II applies as these products are "by nature considered biological medicinal products as the active substances are live microorganisms, which are biological substances" (Cordaillat-Simmons et al., 2020). The FDA has provided guidance for LBPs via the guideline for "Early Clinical Trials With Live Biotherapeutic Products." In a similar way, the EMA has provided a monograph. However, none of the regulatory frameworks specifically address LMPs administered by injection. This is precisely where the topic gets challenging. In the European Union, a drug product intended for administration by injection would commonly fall under the scope of EudraLex Volume 4–Annex 1—"production of sterile medicinal products." However, inherent to the nature of LMPs, those products are not sterile. They might be considered "monoseptic" in the case of single-strain products or even contain a multitude of different live bacterial strains in the case of multistrain or consortium-based products. On the other hand, the fact that LMPs are administered into the bloodstream, injected directly into solid tumors, or applied to open wounds in patients who might inherently be considered "vulnerable" requires that these LMPs are free of any microbial or viral contaminations. Bioburden-controlled production processes, typically governed by EudraLex Volume 4–Annex 2, are not deemed sufficient to ensure patient safety. On the other side, a strict adherence to EudraLex Volume 4–Annex 1 might not be technically feasible, due to the nature of the typical production processes of live biological products. The remaining question is whether regulatory bodies, sponsors, and service-providing companies are aligned on this topic, given the lack of currently approved LMPs administered via injection. Hence, this paper intends to provide a basis for approval of such injectable LMPs in the future by considering the current landscape of clinical trials and regulatory guidelines.

## 3 Overview of current clinical trials involving live microbial products

In 2020, according to Servetas et al. (2022), there were 134 active clinical trials involving LMPs. In 2025, we retrieved 153, excluding all clinical trials that we considered involving true FMTs (**Supplementary Table 1**). The increasing interest in using LMPs as therapeutic agents results in experiences that can be used not only to further refine this niche regulatory landscape but also to learn from experience.

A clinical trial database—ClinicalTrials.gov—was searched using the keywords "Anaerobic" (excluding "Exercise," "Muscle," and "Performance"), "Microbial AND LBP," and "Solid Tumor AND Microbial." The datasets retrieved were manually searched for relevant trials, excluding all trials that were of "observational intent," focused on FMTs, or involved probiotics. Finally, the dataset was completed by searching for specific products known from the literature, resulting in a total of 153 clinical trials (**Supplementary Table 1**). To simplify categorization and allow for clustering, detailed target descriptions were replaced by "cancer/solid tumors" or "infections/chronic disease" where possible. Bacterial strain nomenclature was also simplified. The curated result of the search (**Supplementary Table 1**) is a non-exhaustive list of the current clinical trial landscape.

**Figure 2:**
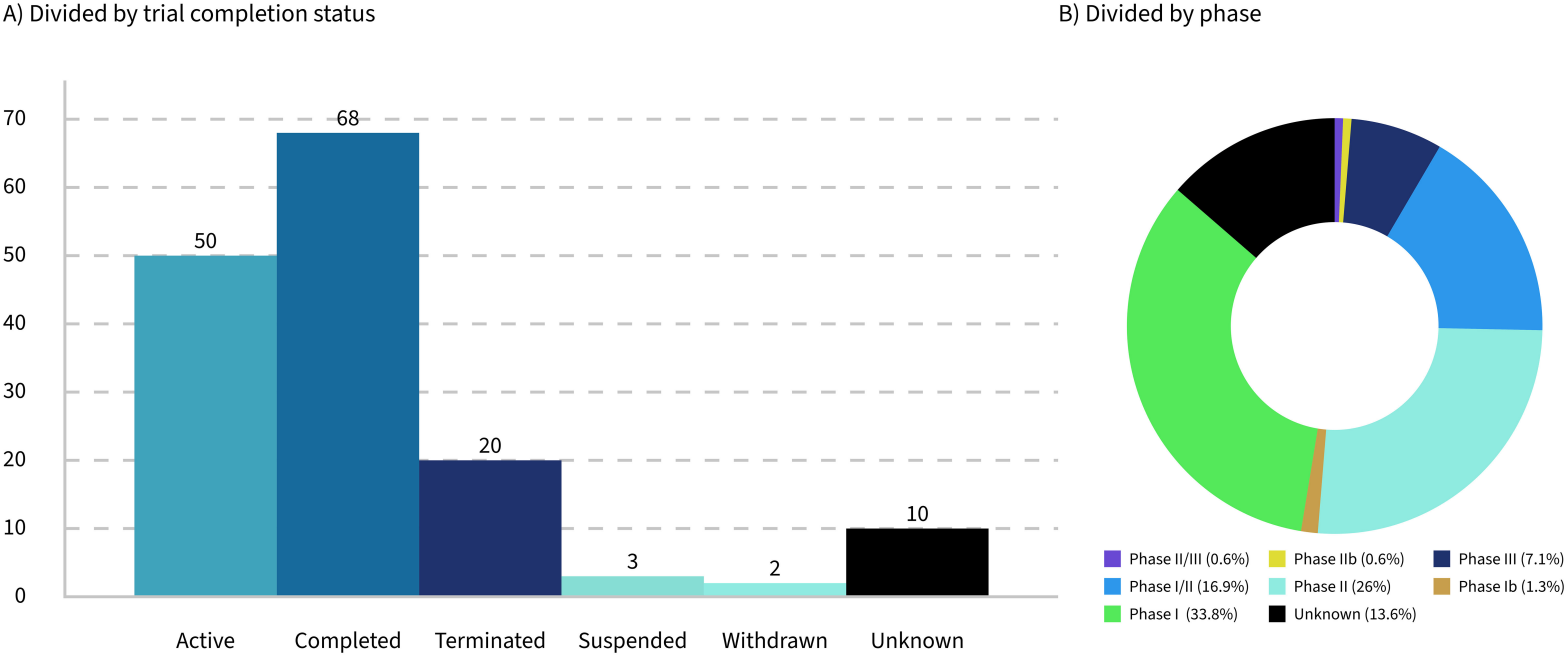
Breakdown of 153 LMP clinical trials by completion status **(A)** and phase **(B)**.

Only 32% of the trials analyzed are currently active, while 16% have been terminated, suspended, or withdrawn (**Figure 2A**). These figures highlight significant challenges faced by the trials and their sponsoring companies, such as feasibility issues, funding shortages, recruitment difficulties, and unforeseen complications that hinder progress or completion. The low percentage of active trials suggests that many have either concluded or failed to progress. This can contribute to a biased perception of success, particularly given that the progress and outcomes of the trials are often underreported.

Seventy-eight percent of the trials identified in this study were between phase 1 and phase 2 (**Figure 2B**), reflecting the early stage of development in the field of LMPs. This predominance of early-phase trials highlights the experimental nature of these products and the challenges associated with their development. However, it is important to note that the lack of updates on trial progress in databases such as ClinicalTrials.gov may have influenced the dataset, potentially underestimating the number of trials that have advanced to later phases.

If we look at the number of trials in categories as per Section 2.1, the majority of products in clinical trials are LMPs; only four different products involve inactivated organisms (inactivated, non-pathogenic, gram-negative bacteria in one case; *Prevotella histicola* in the other case; and *Mycobacterium obuense* in the last two cases). Of those four, only the *P. histicola* product is orally administered.

Thirty-six trials were identified as clearly involving engineered strains. Of these, only 11 are administered orally, while the remainder are either applied topically or administered via injection (**Figure 1B**).

Out of the 153 clinical trials in the present dataset, 103 trials are based on products that are either orally or topically (including vaginally or rectally) administered. Thirty trials^1^ are based on products that are injected intravenously, intramuscularly, intratumorally, and intradermally or involved any other kind of injection or infusion. Among those that are injectable, only three trials, or 10%, were categorized as vaccines. Products that are intended for administration by injection seem to be prevalently aiming at cancer, particularly solid tumors, with only four known clinical trials involving treatments of other diseases.

Only a very small number of those trials are of a preventive nature (<6%), as the majority of the current clinical trials focus on therapeutic aspects. While most applications of LMPs still focus on different recurrent infections, inflammation, and metabolic disorders (Heavey et al, 2022), 39% of the clinical trials target a form of cancer. This is not surprising for two reasons. First, there are unmet needs as traditional cancer therapeutics do not sufficiently address heterogeneous tumors (Sieow et al., 2021). Combination therapies where LMPs are one component can be an approach. Second, LMPs are perfectly suited as anticancer agents, specifically for solid tumors, as LMPs can survive and act in a specific tumor microenvironment (Steidler et al., 2003; Sieow et al., 2021). LMPs, specifically engineered ones, have the advantage of being naturally tumor-targeting while inherently displaying proinflammatory properties (Charbonneau et al., 2020). This combination gives them a huge potential for future cancer treatments.

Cancer-targeting LMPs are often engineered strains that are administered by injection. The regulatory landscape for this kind of product is the most complex one. However, regardless of whether the microbial product is live or inactivated, non-modified, or engineered, the actual mode of action (**Table 3**) might not always be completely known or fully "deciphered" as phrased by Paquet et al. (2021), rendering not only the clinical study design more challenging but also the establishment of the right testing regime for the manufacturing process difficult. Taken together, this paper proposes a set of classifications for LMPs that clarifies the (regulatory) communication and discussion in the field going forward. If the proposed classification was applied to the dataset at hand, the landscape of the current clinical trials would reflect the previous sections: a large majority of LBP, a minority of vaccines and LBTs, and a growing part of engineered LMPs, represented by the category "MVGT," often targeting tumors (**Figure 1A**).

**Table 3:** Possible mode of action of therapeutic live microbial products.

1. Microbiological (e.g., competitive exclusion)
2. Physiological (e.g., production of short-chain fatty acids)
3. Metabolic (e.g., cross-feeding with other member of the microbiota or production of antimicrobial metabolites)
4. Immunological: • Stimulation (e.g., stimulation of natural killer cells, secretory IgA, or regulatory T cells) • Payload delivery and expression (e.g., CD47, nanobodies, activation of the innate immune system)

## 4 Life cycle for live microbial products: from development to market access

Regardless of the classification, all microbial products for human use fall under regulations related to GMP-compliant manufacturing. This includes the EMA's directive 2001/83/EC, ICH Q11, and the FDA's guidance on process validation (European Commission, 2022; Official Journal of the European Communities, 2001). In this regard, the manufacturing of microbial products is equal to the manufacturing of biological products. Nevertheless, there are live microbial specific challenges that arise in the manufacturing process development when taking these guidelines into account.

While the guidelines and common perception often foresee a linear trajectory—where a process is first developed and characterized, then scaled up to provide material for all clinical phases in the same manner before undergoing process performance qualification—the reality looks slightly different. Indeed, often there are several years between the GMP production of the material for clinical phase I and the process performance qualification batches. During these years, process characterization at a small scale, adaptation of the production process at the final scale, and further development of analytical methods take place. Such data and knowledge, sometimes even arising from deviations encountered at the production scale, can be leveraged to reduce the uncertainty around the linkage between process variables and critical quality attributes. It is important to define early on—and continually refine over the course of the clinical studies—the critical quality attributes (CQAs) via a quality target product profile (QTPP). By linking these CQAs to process variables, their impact can be ranked in terms of severity. Additionally, the initial likelihood of these attributes falling outside of the control space should be assessed, along with the detection mechanisms in place to detect such failure events. This assessment can be further refined throughout the course of process characterization, eventually leading to a defined control strategy that can be put to the test in the process performance qualification stage (stage 2 of process validation according to the FDA guideline), a pivotal step prior to regulatory filing and approval of a new product. This approach has proven to be suitable for a variety of large-molecule products, such as recombinant proteins, conjugated vaccines, or mRNA-based products, and can be translated as such to LMPs, particularly products intended for administration via injection.

When looking at the expected QTPP of an LMP, typical safety-related CQAs include the absence of contaminants and proof of monoseptic condition of the final product. Based on the CBER Guidance for Industry: Early Clinical Trials with Live Biotherapeutic Products: Chemistry, Manufacturing and Control Information (February 2012) and Recommendations for Microbial Vectors used for Gene Therapy (September 2016) as well as the European Pharmacopoeia monograph 3053, the approach to testing LMPs (in terms of sterility) intends to demonstrate a monoseptic product by multiple purity tests (e.g., microbial limit test or absence of specified organisms as defined in the Pharmacopeia), rather than by a single sterility test. It is important to highlight that the development of methods is difficult, as the LMP itself, typically present in high concentration, may interfere in many ways, for instance, with the detection of other organisms.

### 4.1 Manufacturing and analytical challenges for monoseptic products

**Figure 3:**
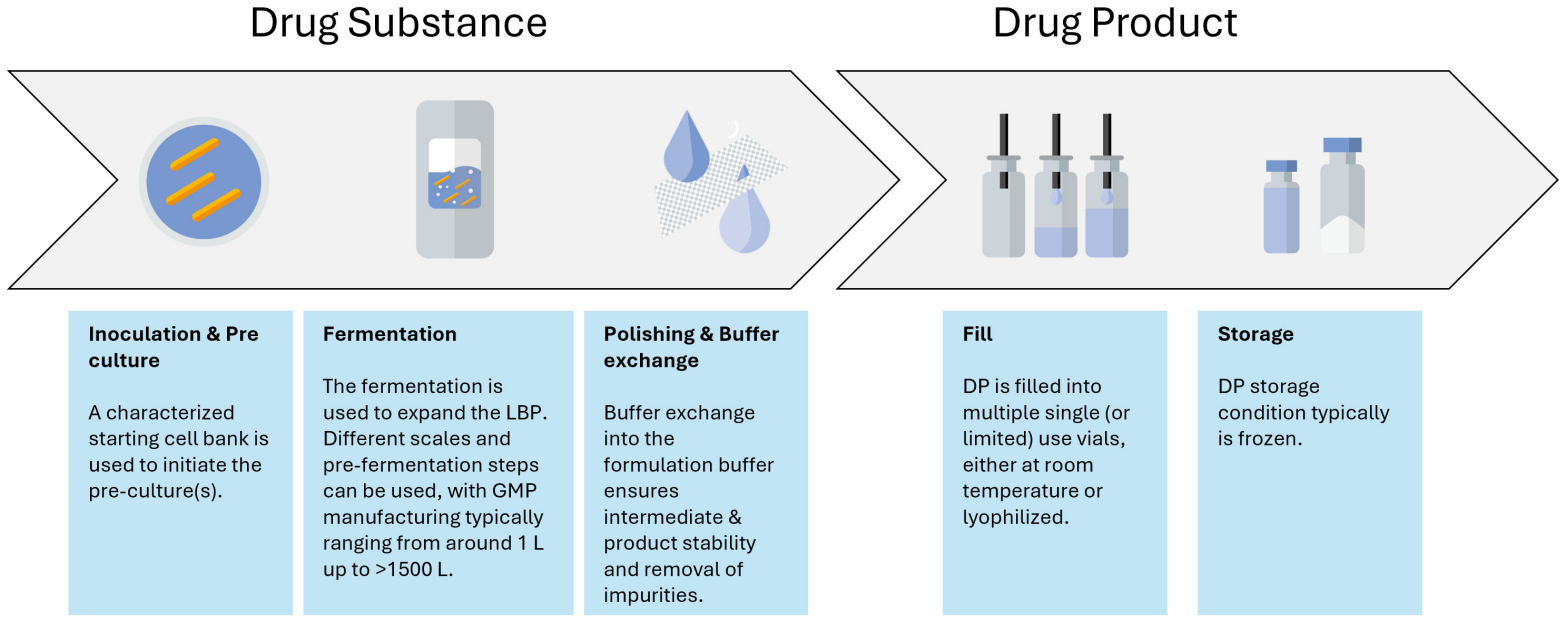
Manufacturing process flow as typically used for the manufacturing of LMPs.

The aim during development is to go from a typical discovery stage process to a large-scale manufacturing process. In contrast to biological products, the master cell bank is already the API. A typical LMP drug substance manufacturing process has a very simple downstream process focusing mainly on polishing and buffer exchange of the LMP intermediate substance compared to the biologics field where there is a need to isolate a specific biological entity from the cell debris (**Figure 3**). Hence, a more thorough characterization is expected from a regulatory point of view for LMPs' starting material, as these cell banks might directly influence the quality of the final product by means of aspects related to safety and efficacy (Paquet et al., 2021). It is usually expected that the origin of the strain(s) is documented, and the passage history leading to the creation of a research cell bank (RCB), master cell bank (MCB), or working cell bank (WCB) is described (Paquet et al., 2021). Clinical phase I trials can still be carried out with MCBs, but it is advised to start using and characterizing WCBs as early as possible to preserve the MCB stock. A change in cell bank after clinical phase I will result in a more extensive comparability exercise than for biologics manufacturing (Levine et al., 2017).

The manufacturing process itself also requires special attention. It needs to be developed into a process where every unit operation is performed in a closed system, seamlessly integrating with the next unit operation to prevent the introduction of viral or bacterial contamination. This is especially important for LMPs, as it is impossible for these samples to undergo holistic microbial evaluations. Therefore, the manufacturing control strategy often relies on different single-use disposables or confirmed clean multipurpose equipment together with closed systems (such as, for instance, a Thermo Fisher eSUF) that are connected to each other via different aseptic connections. Those can be either commercially available systems (such as AseptiQuik^®^ or ReadyMates^®^), welds of different tubes, or via a controlled piping system. Additionally, the contamination control strategy often contains monitoring and product-specific testing. Inactivated LMPs might bear the inherent advantage of the possibility to perform sterility testing; however, they require the development of a specific inactivation assay to testify that the organism is indeed inactivated at the end of the relevant unit operation. Altogether, the contamination control strategy ensures a monoseptic end product by prevention and good design, as well as controls and monitors the manufacturing process supplemented by analytical evaluations (**Figure 3**).

Another important aspect to consider during the LMP manufacturing process development and scale-up is the formulation of the cell harvest. Formulation provides stability over the unit operations, such as filling, freezing, and lyophilization, as well as over the intended shelf life. Although non-frozen storage, either chilled or at room temperature, can be considered, freezing of the LMP is often required for longer-term shelf life. The impact of freezing and thawing needs to be explored during process characterization. Indeed, viability is likely a CQA of any LMP. As such, any aspect affecting viability can potentially be considered a critical process parameter (CPP). In a commercial process, the control strategy associated with any CPP needs to be robust and reliable to yield an efficacious product. Lyophilization can be one of the options for controlled freezing associated with drying. Controlled freezing to –20°C or −70°C using controlled freezing systems such as the RoSS systems from SUS or the Celsius CTF systems from Sartorius can also be options, while other products that are currently in clinical trials are based on simple freezing of the drug product in conventional freezers as it is known from the biologics field. Regardless of the approach to freezing, the physiological state of the cells prior to the unit operation, in combination with the right formulation during the unit operation, will influence the viability of the cells upon reconstitution and hence directly impact the CQA "content" (Carvalho et al., 2004; Muñoz-Rojas et al., 2006).

The use of a suitable cryoprotectant is particularly important as it plays a vital role in minimizing the loss of viability when freezing the cells. Commonly used cryo- or lyoprotectants include saccharides, polyols, and amino acids or proteins (Grilc et al., 2023). As disaccharides are known as effective cryo- or lyoprotectant (Muñoz-Rojas et al., 2006), it is not unexpected that trehalose is one of the most often chosen cryoprotectants. Non-penetrating cryoprotectants such as trehalose reduce both intra- and extracellular ice formation (Bircher et al., 2017) by influencing osmotic effects, whereas polyol cryoprotectants such as glycerol reduce intracellular ice formation by acting on the properties of the cell membrane (Hubálek, 2003; Fowler and Toner, 2005; Bircher et al., 2017). LMPs contain live cells; hence, it is important to use non-fermentable sugars to prevent cell growth and acidification.

Whether orally or otherwise administered, choosing the right formulation is critical for all types of LMPs. The survival of the active ingredient (the living cells) and hence the clinical efficacy of orally administered LMPs depend not only on the cells' intrinsic, natural resistance to the potentially harsh manufacturing (e.g., harvest) and storage conditions but also on the matrix in which the cells are formulated (Pot and Vandenplas, 2021). These do not only have to pass the gastrointestinal tract but must also be able to survive and proliferate in those environments, competing with the microbiota already present (Heavey et al, 2022). In the case of oral LMPs, there is often a capsular formulation, adapted to the needs of maintaining the viability of cells throughout the passage through the gastrointestinal tract. Those capsular formulations are also often carbohydrate-based (Pot and Vandenplas, 2021).

For LMPs administered via injection, liquid formulations (or reconstituted liquid formulations) must be designed to prevent aggregate formation and maintain a homogeneous suspension while protecting against harsh conditions of lyophilization. This ensures shelf-life stability of the products.

As those cryo- and lyoprotectants, as well as any other formulation components, are considered excipients, it is important that they comply with the guidelines regulating excipients. In this context, it is important to control the endotoxin content of the excipients, as endotoxin testing can be complicated by the presence of the LMP API. In addition to freezing, cooling can also be considered, potentially in combination with reconstitution after freeze-drying of the API.

Assessing viability is crucial for in-process testing to gain process knowledge during development. One of the challenges associated with freezing is the loss of viability observed upon reconstitution or thawing. This loss of viability presents two challenges. First, while ideally minimized, loss of viability is often an uncontrolled and poorly characterized phenomenon, making it difficult to manage in a robust and reliable manner. Second, the assumption that the loss of viability is understood, characterized, and controlled to be reproducible adds another layer of complexity. Additionally, it is widely recognized in the industry that bulk lyophilization, a common unit operation in the manufacturing of LMPs, poses significant challenges in terms of sampling.

The challenge is further amplified by the absence of meaningful and reliable analytical methods for in-process control. Often, total cell count, a fast and reliable method, is used as an indicator of in-process testing or in-process control. For LMPs, viability is, almost by definition, a critical quality attribute. Typically, viability is assessed via plate-based (spread or pour plate) or cell counter methods of cell enumeration. However, at the drug substance and drug product levels, total cell count is then often paired with any viable cell count method. Both the viable cell count and the total cell count methods, as well as dielectric spectroscopy or any other such method, are often used in the industry, while primary research and development use tools such as metabolic profiling (Kim et al., 2022) or genotypic analysis (Paquet et al., 2021). Although the industry might benefit from bringing such latter methods to a level where they can be used in routine quality control, the complexity of the methods and the difficulties with ensuring appropriate intermediate precision might currently still hinder their implementation.

Zaragoza and colleagues provided a clear overview of six key categories of analytical assays commonly used to evaluate the release profiles of microbial products. The first category involves growth curves, typically derived from cell count methods, which serve as a fundamental tool. The second category, flow cytometry, enables high-throughput analysis during the development phase. Similarly, the third category, colorimetric assays, also supports high-throughput workflows but may offer a more cost-effective alternative in terms of equipment investment. Spot assays, the fourth category, are appealing due to their simplicity, requiring neither specialized equipment nor highly trained personnel. However, their precision may fall short for later stages of development and manufacturing. The fifth and sixth categories, fluorescence microscopy and transcriptomics, provide valuable insights but are often limited by their high costs, time demands, and challenges in qualification (Zaragoza et al., 2024).

Once developed, the manufacturing process will be put to the test during the second stage of process validation. Assessing the consistency of the manufacturing process, particularly batch-to-batch consistency, is key to a successful process performance qualification (PPQ) campaign as required for market access. For example, variations in the quantity of live microorganisms between batches might be greater than variations expected in the production of biologics. The substantial variation in cell counts and viability evaluation methods makes it challenging to develop meaningful control strategies. Hence, it might be tempting to broaden the product specification (Paquet et al., 2021). However, most importantly, product specification in terms of cell counts (e.g., CQA "content") needs to be properly justified and fit the overall picture of manufacturing consistency and product requirements (also quote Cordaillat-Simmons et al., 2020).

Furthermore, depending on the physiological state, cells might not be able to divide and form colonies but may "nonetheless retain sufficient metabolic activity to perform some engineered functions *in situ*" (Charbonneau et al., 2020). Instead of pour or spread plates, enumeration using live/dead staining might be a more accurate alternative to the currently used cell count and viability testing. However, such methods are also more cumbersome to realize in routine GMP manufacturing. Real-time viability assessment, additionally compatible with single-use equipment, such as dielectric spectroscopy (Dabros et al., 2010), might be an interesting alternative that still has to find its way into routine GMP manufacturing. However, neither dielectric spectroscopy nor live/dead cell enumeration may be appropriate for monitoring the activity of cells in manufacturing processes, where active cell division serves as the actual indication or surrogate measurement of potency. It is important to note that in certain cases, viable cell count is used as a surrogate of potency, whereas a cell-based assay might be used to determine the efficacy.

### 4.2 Extrapolating those challenges to the manufacturing of consortia-based microbial products

**Figure 4:**
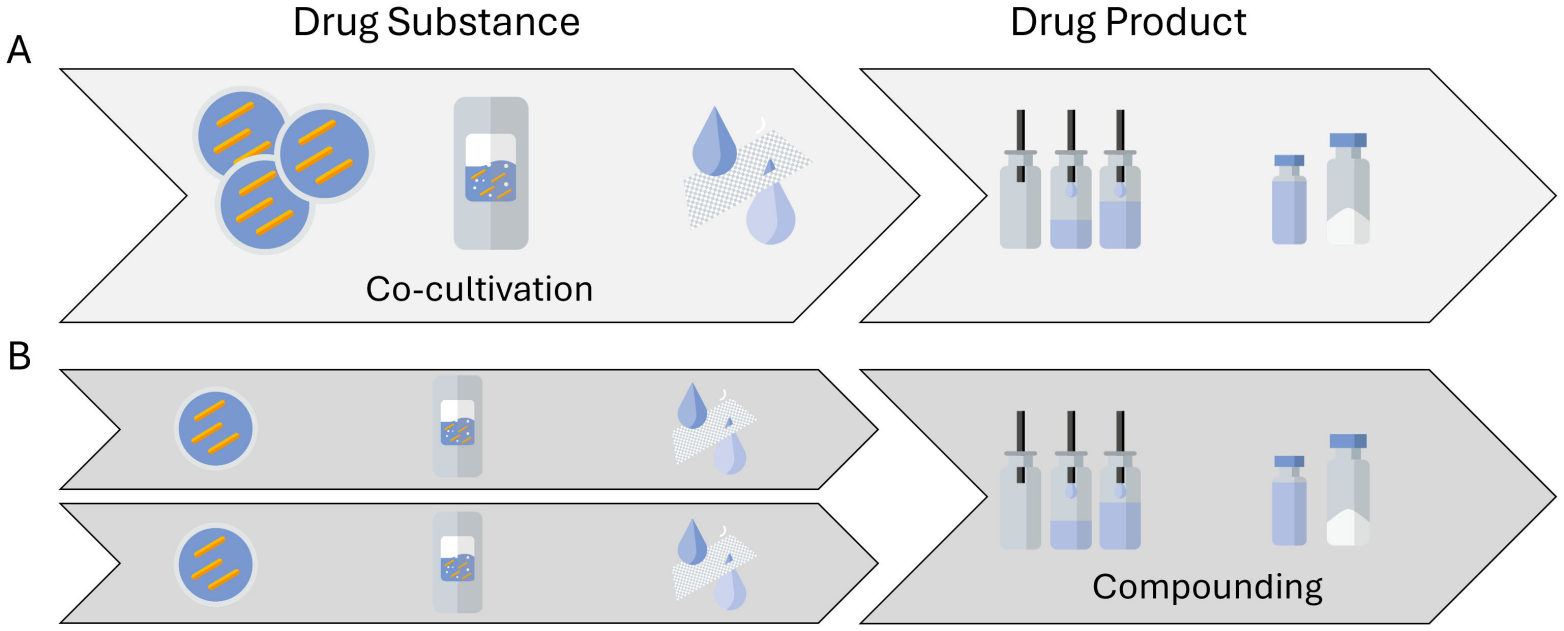
Possible routes to manufacture consortia-based, multistrain live microbial products. **(A)** A multistrain cell bank is used to generate a co-cultivated drug substance and subsequently a multistrain drug product material. **(B)** A single-strain drug substance material is manufactured, after which several different strains are compounded into a multistrain drug product.

The different challenges mentioned above are even greater when shifting focus from single-strain LMPs to consortia-based LMPs. Consortia-based products consist of multiple different strains in the same formulation, thereby increasing the efficacy or broadening the indication of such a therapeutic, if not both. The distinction needs to be made first between LMPs where strains are truly co-cultivated (**Figure 4A**) and those where the different strains are produced and harvested separately and then formulated into a single drug product (**Figure 4B**). True co-cultivation yields an end product in one manufacturing run but reduces control over the individual strain growth profiles. Hence, a co-cultivation approach might be challenging for products where tightly controlled therapeutic manufacturing is a must. It is hence not surprising that only a few entities are engaged in researching and developing true co-cultivation products. A more traditional approach is to grow single strains and thereafter compound them together in a predefined ratio. This is the approach chosen by most of the entities involved in the 46 clinical trials involving multiple strains. However, cultivating strains separately results in multiple batches, which also increases manufacturing time and costs. Furthermore, the metabolome of a co-cultivated consortia might differ from the metabolome of a formulated consortia, where the clinical benefits stem from the nature of manufacturing the next generation of consortia-based products.

In the compounding instance, analytical methods are often developed for the individual strains. The challenges then lie in ensuring that the purpose of the assays is still fulfilled for the product, where the strains are combined. As for the single-strain products, the challenge is often in finding purposeful methods or surrogate methods in the first place. Surrogates of potency and identity for LMPs are often methods around cell counts. However, such cell count methods, whether they are plate methods, optical density measurements, or dielectric spectroscopy-based measurements, are agnostic of the identity of the strains that they measure, meaning that they lack strain specificity to distinguish between a signal coming from a single-strain cell suspension or a multistrain cell suspension. While Magalhães et al. (2019) or Kallastu et al. (2023) are presenting research options to overcome the challenge of content or potency method surrogates for both single-strain and consortia-based live bacterial therapeutics, currently, there seem to be no viable options for routine quality control. Although not commonly applicable for consortia, in specific cases, identities could be distinguished by selective growth media, colony morphology, or other characteristics. Challenges, however, go beyond the appropriate quality control methods for consortia-based LMPs. As there are many advantages in consortia-based LMPs, it is not surprising that several research entities are engaged in developing the co-cultivation field.

## 5 Conclusion and perspectives

Bringing any medicinal product to the market entails going through the whole life cycle of process validation, starting with process development and characterization (also called stage 1 of process validation according to the FDA guidelines), followed by process performance qualification in order to provide documented proof that the process and associated control strategy that was developed are consistently delivering a product meeting predefined specifications suitable to the commercial production of the product. The traditional approach used for the development and characterization of manufacturing processes for biologics is ensuring that the relevant, phase-appropriate understanding is gained while that knowledge is leveraged across the different stages of process validation. The same approach is needed for LMPs, regardless of the category they fall into (**Table 2**). The development and characterization of a manufacturing process for an LMP are impacted by the regulatory category, as different regulations and guidelines need to be fulfilled. Nuances in the requirements for the manufacturing process, the application (therapeutic or preventive), the target (tumor or chronic infection), and the route of administration (injected or oral) are crucial in defining the specificities of the development and validation program.

Ultimately, overarching guidelines such as ICH Q11 and the FDA's guidance for process validation require an approach of continuously evolving documented process and product knowledge generation. This ensures that the process, developed in close collaboration with the authorities responsible for market authorization approval, can consistently produce products of appropriate quality. This is achieved through a data-driven control strategy, which is validated during a process performance qualification campaign.

## Footnotes

1 For the remaining 20 products on trial, no clear information could be retrieved.
